# The Severity of COVID 19 Pneumonia in Vaccinated vs. Non-vaccinated Patients in the Second Wave: An Experience From a Tertiary Care Center in India

**DOI:** 10.7759/cureus.25378

**Published:** 2022-05-26

**Authors:** Jyoti Bajpai, Surya Kant, Ajay Verma, Ajay K Patwa, Virendra Atam, Shyam C Chaudhary, Anuj Pandey

**Affiliations:** 1 Pulmonology, King George's Medical University, Lucknow, IND; 2 Gastroenterology, King George’s Medical University, Lucknow, IND; 3 Neurology, King George’s Medical University, Lucknow, IND; 4 Internal Medicine, King George's Medical University, Lucknow, IND

**Keywords:** oxygen therapy, comorbidities, severity, vaccination, covid 19

## Abstract

Background: Coronavirus disease 2019 (COVID-19) is a novel infectious disease caused by SARS CoV-2 that emerged in Wuhan, China, and has rapidly spread worldwide. The mortality rate of critically ill COVID-19 patients is high.

Objective: To assess the severity, different clinical symptoms, and comorbidities of COVID-19 pneumonia in vaccinated vs. non-vaccinated patients.

Methods: In this single-center, cross-sectional study, 142 patients with COVID-19 were enrolled. The clinical characteristics, comorbidities, severity, and outcomes were also assessed.

Results: Of the 142 patients, 92 (64.8%) were males, with a mean age of (56.00±14.81) years. Among them, 62 (43.7%) were aged above 60 years. Of these, 92 (64.7%) had comorbidities. The patients were divided into two groups: unvaccinated and those who received at least one dose of the vaccine within six months. The demographic characteristics of the two groups were similar except for gender. In the vaccinated group, most of the patients were males. Most patients in the non-vaccinated group had a severe illness, whereas most patients in the vaccinated group had mild to moderate disease. Only 26% of the vaccinated group experienced severe illness compared to 71.5% in the unvaccinated group. In addition, the all-cause 30-day mortality in the non-vaccinated population was higher than that in the vaccinated population. However, this difference was not statistically significant (12.5% vs. 7.1%). On the contrary, there was no difference in the length of the intensive care unit or total hospital stay between the two groups.

Conclusion: Severe COVID-19 had the worst outcome in the unvaccinated patients. Most partially vaccinated patients got infected before developing immunity, and a small percentage of completely immunized patients who were infected were likely non-responders. Receiving at least one vaccination dose significantly reduced illness severity.

## Introduction

As of December 12, 2021 [1], the total number of cases of coronavirus disease 2019 (COVID-19) had surpassed 200 million, and the death toll has exceeded 4 million worldwide since the start of the COVID-19 infection and its declaration as a pandemic on March 11, 2020 [2]. Despite the incredible efficiency of the COVID-19 vaccine, the infection still occurs in vaccinated patients, albeit with a milder severity than in unvaccinated patients.

As no vaccine is 100% effective, some vaccinated individuals get infected [3]. However, it is expected that these individuals will have a less severe form of infection, less frequent hospitalization, and intensive care unit (ICU) admission. This is possibly achieved through the vaccine's ability to produce immunological memory responses, which hastens the onset of infection [4,5]. Several investigations of patients infected with COVID-19 after vaccination corroborate this hypothesis.

The vaccine's effectiveness in preventing hospitalization after the second dose was 87% in a case-control trial. Prospective cohort research has shown that COVID-19 immunized cases have a shorter duration of illness and a decreased risk of infection [6].

Most research on post-vaccination hospitalized COVID-19 cases analyzes the severity of the disease using ICU admission, mechanical ventilation (MV), and fatality as criteria [7,8]. However, to the best of our knowledge, no study has attempted to determine the severity of COVID-19 infection among vaccinated patients; therefore, this study was conducted based on the hypothesis that vaccinated patients admitted to the hospital may have a lower disease severity than unvaccinated patients.

## Materials and methods

This prospective observational study was performed at King George Medical University. A total of 142 patients were enrolled in the study between April 2021 and July 2021. COVID-19 diagnosis was confirmed by a reverse transcription-polymerase chain reaction of nasopharyngeal swabs in adults (age ≥18 years). We excluded pregnant women, patients with active pulmonary tuberculosis, and those who tested positive for the Human Immunodeficiency Virus. The Institutional Ethics Committee approved this study. Written and informed consent was obtained from all patients enrolled in the study.

Baseline demographic and chronic health characteristics were collected from all recruited patients (age, sex, occupation, clinical symptoms, and comorbidities). Additionally, we recorded vital signs, peripheral oxygen saturation on room air (SpO2), supplemental oxygen needed to maintain SpO2 at 94%, and the respiratory rate upon admission to the hospital. Mild symptoms and no dyspnea were categorized as mild illness; moderate illness was defined by the presence of clinical features of dyspnea and or hypoxia, fever, cough, including SpO2 90 to ≤93% on room air, Respiratory Rate more or equal to 24 per minute with pneumonia with no signs of severe disease and severe illness is characterized by clinical signs of severe pneumonia plus one of the following respiratory rate >30 breaths /minute, severe respiratory distress, SpO2<90%. This allowed patients to be classified as having mild, moderate, or severe disease according to their severity at hospital admission [9]. Enrolled patients were followed up during their hospital stay. Initially, we divided the patients into three groups based on their vaccination status (no immunization, single-dose, and two doses). However, by the end of the study period, we enrolled only three patients who received two doses of vaccination; therefore, we divided the patients into two groups: no vaccination or at least one dose of vaccination to avoid the statistical disadvantages of a small group [10]. 

Steroids, antivirals, and tocilizumab were among the treatment factors identified in the general drug categories. Finally, each patient's 30-day outcome was recorded as dead and discharged and the length of stay in the hospital. Patients who were sent to other hospitals were not tracked and were treated as alive and dismissed from our facility.

The primary goal of this study was to compare the severity of COVID-19, clinical symptoms, comorbidities, and inflammatory markers between vaccinated and non-vaccinated patients. The 30-day all-cause mortality and need for MV at ICU admission were secondary outcomes.

Statistical analysis

Continuous variables are summarized as the mean and standard deviation, and continuous variables were compared between groups using the Student's t-test. In contrast, discrete variables are summarized as frequencies and percentages (percent) and were compared between groups using the chi-square test. All statistical tests were two-tailed and considered significant at p <0.05. Statistical tests were performed using commercially available software, SPSS (Statistical Package for Social Sciences) Version 21.0.

## Results

There were 162 admissions to the ICU with a confirmed COVID-19 diagnosis during the study period. Twenty patients were excluded, leaving a total of 142 enrolled patients. Pregnant females, lactating women, and patients who did not consent were excluded from the study. The study population comprised 142 participants aged between 18 and 83 years with a mean age of 56.00±14.81 years. Most of the patients were aged >60 years (43.7%). The sex ratio in our study population showed that the number of males was higher than that of the females, i.e., 64.8% and 35.2%, respectively (Table 1). 

**Table 1:** Comparison of demographic variables (Age and sex) between the vaccinated and unvaccinated groups

		Vaccination Status				p-value
		Vaccinated		Unvaccinated		
		N	%	N	%	
Age intervals	18 to 45 years	1	5.30%	37	30.10%	0.031
	46 to 60 years	5	26.30%	37	30.10%	
	Above 60 years	13	68.40%	49	39.80%	
Sex	Male	16	84.20%	76	61.80%	0.057
	Female	3	15.80%	47	38.20%	

**Figure 1:**
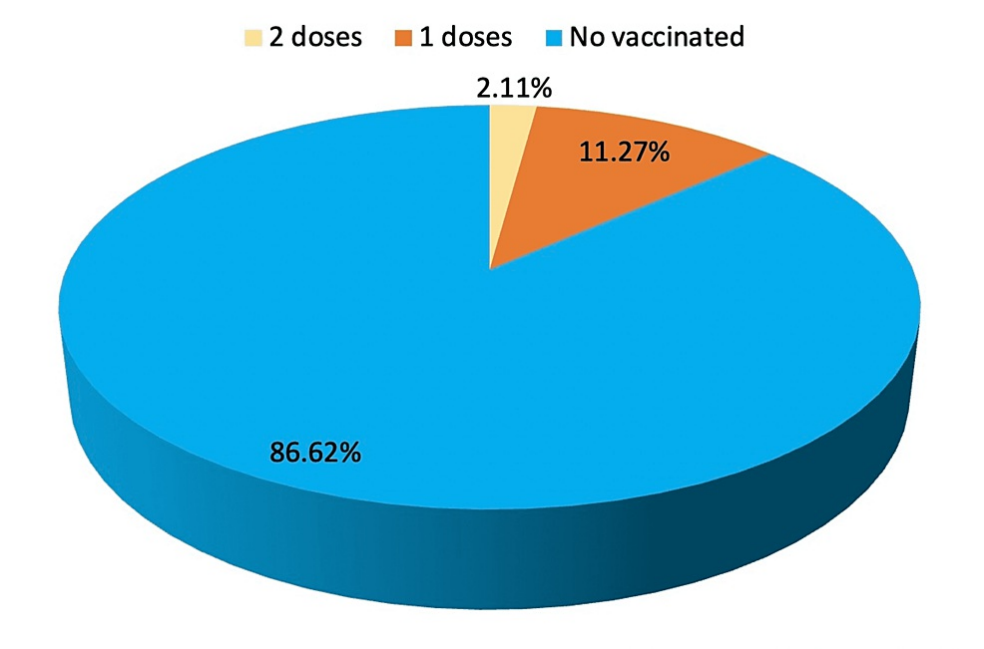
Status of vaccination in hospitalised COVID 19 patients

Only three individuals received two vaccine doses, whereas 123 (86.6%) did not receive any (Figure 1).

Patients were divided into two groups: unvaccinated and those who received at least one dose of the vaccine within six months. The demographic characteristics of the two groups were similar except for gender. In the vaccinated group, most of the patients were males (16 {84.2%} vs. 76 {61.8%}, n=0.057). The vaccination status was significantly higher in the older age group (>60 years) than in the non-vaccinated group (68.4% vs. 39.8, p=0.034). Most patients in the non-vaccinated group had a severe illness, whereas most patients in the vaccinated group had mild to moderate disease. Only 26% of the individuals in the vaccinated group experienced severe illness (Table 2).

**Table 2:** COVID 19 Severity among vaccinated and unvaccinated groups

COVID SEVERITY	Vaccination Status				p-value
	Vaccinated		Not vaccinated		
	N	%	N	%	
Severe	05	26.3%	88	71.5%	0.847
Mild + Moderate	14	73.7%	35	28.5%	

Although the percentage of patients who required MV upon ICU admission was higher among the non-vaccinated patients than in the vaccinated patients; however, the difference was not statistically significant. In addition, the all-cause 30-day mortality in the non-vaccinated group was higher than that in the vaccinated group; however, the difference was not statistically significant (12.5% vs. 7.1%) (Table 3).

**Table 3:** Comparison of Outcome between both the groups

Outcome at 30 days	Vaccination Status						p-value
	Vaccinated		Not vaccinated		Total		
	N	%	N	%	N	%	
Expired	1	5.2	13	9.1	14	9.8	0.465
Survived	18	94.8	109	90.9	127	90.2

On the contrary, there was no difference in the ICU or hospital stay length between the two groups. Fever, dyspnea, and cough were the most common symptoms in both groups; however, hemoptysis and taste alteration were significantly higher in the vaccinated group. The most common comorbidities were diabetes mellitus (DM) and hypertension (HTN) (Table 4).

**Table 4:** Clinical symptoms and comorbidities between vaccinated and unvaccinated group CAD-Coronary artery disease, HTN-Hypertension, DM-Diabetes Mellitus, COPD - Chronic obstructive pulmonary disease, ILD- Interstitial Lung Disease, Post TB- Post-Tuberculosis, CKD-Chronic Kidney Disease, CLD - Chronic Liver Disease, VTE- Venous Thromboembolism, PE- Pulmonary Embolism, H/O ATT-History of anti-tubercular treatment, SBP- Systolic Blood Pressure, DBP- Diastolic Blood Pressure, PR-Pulse Rate, RR- Respiratory rate, Spo2 - Oxygen Saturation

Symptoms, Comorbidities	Vaccination Status					p-value
	Vaccinated			Not vaccinated		
N		%	N	%
DYSPNEA	17	89.5%		102	82.9%	0.471
COUGH	15	78.9%		96	78.0%	0.930
FEVER	18	94.7%		110	89.4%	0.470
HEMOPTYSIS	5	26.3%		10	8.1%	0.016
ANOSMIA	6	31.6%		21	17.1%	0.134
ALTERATION OF TASTE	6	31.6%		13	10.6%	0.012
CONJUCTIVITIS	1	5.3%		4	3.3%	0.581
HEADACHE	1	5.3%		7	5.7%	0.940
OTHER SYMPTOMS	2	10.5%		5	4.1%	0.236
CAD	0	.0%		3	2.4%	1.000
HTN	10	52.6%		29	23.6%	0.008
DM	13	68.4%		47	38.2%	0.013
HEART FAILURE	1	5.3%		8	6.5%	0.836
COPD	2	10.5%		5	4.1%	0.236
ASTHMA	1	5.3%		3	2.4%	0.441
ILD	4	21.1%		1	.8%	0.001
POST TB	1	5.3%		1	.8%	0.251
CKD	2	10.5%		1	.8%	0.047
CLD	1	5.3%		0	.0%	0.134
VTE/PE	0	.0%		0	.0%	NA
H/O ATT INTAKE	1	5.3%		0	.0%	0.134
ANY MALIGNANCY	0	.0%		0	.0%	NA
ANY CONNECTIVE TISSUE DISEASE	0	.0%		0	.0%	NA
Obesity	3	15.8%		12	9.8%	.426
H/o contact covid cases	8	42.1%		19	15.4%	0.006
Smoking	5	26.3%		7	5.7%	0.003
H/o Immunosuppressive therapy	2	10.5%		1	.8%	0.047
SBPmmHg	131.44	14.26		123.40	12.72	0.021
DBPmmHg	76.56	10.98		78.11	8.16	0.500
PR(/min)	88.79	13.37		97.62	81.83	0.689
RR(/min)	23.29	3.95		26.40	21.07	0.584
Spo2 in room air	90.33	3.85		89.17	7.89	0.575

In addition, several individual comorbidities such as heart failure, lung diseases, chronic obstructive pulmonary disease, asthma, and post-tuberculosis sequelae were also prevalent in the groups (Table 4). Eight (42.1%) vaccinated patients had a history of contact covid. However, the 19 (15.4%) cases had a history of contact covid cases in non-vaccinated patients. The number of smokers was higher in the vaccinated group, and this difference was significant (26.3% vs. 5.7%, p=0.003). Most of the patients in the vaccinated group received immunosuppressive therapy (10.5% vs. 0.8%, p=0.04). Vaccinated patients had lower inflammatory markers such as serum C-reactive protein (CRP), ferritin, pro-brain type natriuretic peptide (BNP), and procalcitonin. However, this result was only significant for CRP (Table 5).

**Table 5:** Comparision of various Laboratory and radiological parameters in both group Hb- Haemoglobin, TLC- Total Leucocyte counts, S.CRP- Serum C reactive protein, S LDH- Serum lactate dehydrogenase, Pro BNP- pro B type natriuretic peptide, S PCT -Serum procalcitonin, SGOT -Serum glutamic oxaloacetic transaminase, RBS-Random blood sugar, ABG-Arterial blood gas, PCO2- partial pressure of carbon dioxide, PO2-partial pressure of Oxygen, HCO3-Bicarbonate, Na-Sodium, S.K-Serum potassium, S.Ca -Serum calcium, CT-Computer tomography

Laboratory Parameters	Vaccination Status					
	Vaccinated		Not vaccinated		P-value	
	Mean	SD	Mean	SD		
Hb g/dl	12.43	1.94	12.50	2.69	0.917	
TLC cells/mm^3^	4529.47	3374.12	3858.88	4046.34	0.494	
Platelet count cells/mm^3^	1.86	.64	1.98	1.62	0.749	
S.CRP mg/L	73.74	74.4	904	360	0.011	
S.ferritin mcg/L	503.48	459.60	844.31	1383.52	0.290	
S.LDH IU/L	1068.67	319.66	1098.34	933.01	0.891	
Pro-BNP pg/ml	460.16	402.76	646.54	831.94	0.340	
S.PCT ng/ml	.65	.69	1.23	1.79	0.162	
S.creatinine mg/dl	1.8	3.43	3.25	1.89	0.030	
S.Urea mg/dl	49.18	39.01	47.66	38.94	0.878	
S.Bilirubin mg/dl	1.29	2.38	1.94	2.14	0.637	
SGOT IU/L	91.94	144.91	53.86	74.68	0.090	
RBS mg/dl	284.49	125.71	140.45	133.42	0.002	
ABG-PH	9.36	8.90	8.66	8.47	0.740	
PCO2	32.27	13.72	40.07	15.31	0.038	
PO2	59.63	10.62	52.57	11.76	0.015	
LACTATE	1.38	.35	6.27	14.39	0.142	
HCO3-	26.19	3.44	28.68	4.69	0.028	
Serum Na mmol/L	134.71	4.11	133.13	17.54	0.699	
S.K mmol/L	3.73	.68	4.98	11.78	0.646	
S.Ca mg/dl	3.80	.88	3.78	1.40	0.947	
Chest X-ray score	10.74	3.41	10.62	3.92	0.901	
CT severity score	8	14	15.258	4.74	0.608	

Logistic regression analysis of vaccinated patients projected as a dependent on age, sex, hypertension(HTN), Diabetes Mellitus (DM), interstitial lung disease (ILD), chronic kidney disease (CKD), hemoptysis, alteration of taste. All parameters were significantly associated with higher odds. In the multivariate analysis of all parameters, only ILD was significant.(Table 6).

**Table 6:** Univariate and multivariate analysis of variables on the outcome HTN-Hypertension, DM-Diabetes Mellitus, ILD- Interstitial lung disease, CKD -Chronic Kidney Disease

Parameters	Unadjusted OR (95% CI)	p-value	Adjusted OR (95% CI)	p-value
Age (>60 years)	3.27 (1.17-9.19)	0.024	0.268(0.067-1.070)	0.062
Sex	3.30 (0.91-11.93)	0.069	-	
HTN	3.60 (1.34-9.71)	0.011	1.759(0.418-7.413)	0.441
DM	3.50 (1.25-9.85)	0.017	2.090(0.550-7.950)	0.279
ILD	32.53 (3.41-310.52)	0.002	52.839(3.898-716.274)	0.003
CKD	14.35 (1.23-166.91)	0.033	1.141(0.021-61.142)	0.948
HEMOPTYSIS	4.036(1.205-13.515	0.024	1.537(0.208-11.352)	0.674
ALTERATION OF TASTE	3.905(1.268-12.032)	0.018	3.095(0.576-16.645)	0.188

## Discussion

The second wave of COVID-19 was dreadful and fearful in India. The second wave began in March 2021, just after the start of the vaccination drive. COVID-19 severity was highly high and affected almost every family in India. The outbreak of the second wave was sudden. The vaccination drive began on January 16, 2021, for healthcare workers and individuals aged ≥60 years. After a month and a half of the vaccination drive, the second wave began; therefore, the number of vaccinated patients in our study was lesser, with more individuals aged ≥60 years [11].

We examined the data of 142 COVID-19 positive patients admitted to the ICU during the study period. We found a significantly higher prevalence of unvaccinated patients in the group, with only 13.4% of patients receiving at least one dose of the vaccine. This is not alarming, given that both vaccination brands approved in India have already shown remarkable efficacy in randomized controlled trials [12,13]. The vaccinated group differed slightly from the non-vaccinated group, and it included significantly more patients with older age and male predominance with mild to moderate illness. According to the Government of India guidelines, individuals older than 45 years were prioritized in the initial vaccination drive. 

Hemoptysis and taste alteration were the most common symptoms in the vaccinated group in our study. Antoleii et al. reported that sneezing was the most common symptom in the vaccinated group [14]. The severity of the disease was higher in the non-vaccinated group. Most patients in the vaccinated group had mild to moderate disease, although, in our study, the number of fully vaccinated patients was meager. The vaccination drive and the second wave of COVID-19 in India occurred simultaneously; therefore, the number of vaccinated patients was lower in the study. A study from Israel reported that those who received two vaccination doses had decreased post-COVID-19 symptoms [15].

Most patients were hospitalized within 20 days of vaccination with a single dose. During the initial 2-3 weeks after the first dose of the vaccination, the efficacy was much lower than what it was subsequently [16]. HTN, DM, ILD, and CKD were significantly higher in the vaccinated group. A study by Mhawish et al. reported the presence of HTN (38.2%), DM (41.2%), and CKD (11.8%) in the vaccinated group. Age, immunization status, comorbidities, disease severity at admission, MV on admission, DM, HTN, CKD, coronary heart disease, and heart failure or arrhythmia were associated with 30-day mortality [17].

The 30-day mortality was higher in the non-vaccinated group. Immunity develops over time; therefore, the efficacy was significantly lower during the initial 2-3 weeks after the first dose of the vaccine. Around this time, spiked immunoglobulin G (IgG) begins to form [18,19]. Three patients in our sample were fully immunized; however, they presented 21 days after their last immunization dose, concluding that they were non-responders (sometimes referred to as breakthrough infection). As most COVID-19 vaccines rely on the formation of spike IgG antibodies, antigen mutations could reduce their efficacy [20], and new variations, perhaps with antigen mutations, have begun to arise worldwide, possibly including India [21].

Most clinical trials on post-vaccination COVID-19 looked at disease severity in terms of symptomatic or asymptomatic presentation, the number of hospitalizations, ICU admissions, MV, or death events [22,23], concluding that vaccinated patients had a lower severity (depending on the study design). Our study describes the clinical profile, comorbidities, severity, and outcome of all-cause 30-day mortality in the initially vaccinated majority with single-dose vaccination and non-vaccinated moderate to severe cases admitted to the ICU during the second wave of COVID 19. 

Vaccinated patients had lower levels of inflammatory markers, such as serum CRP, ferritin, pro-BNP, and procalcitonin, although the result was only significant for CRP. Most smokers belonged to the vaccinated group. Vaccinated patients belonged to high-risk groups; therefore, smoking, immunosuppressive therapy, and contact history were higher in the vaccinated group. The vaccination drive also prioritized older and high-risk individuals with comorbidities. In the unadjusted logistic regression analysis, older age, HTN, DM, ILD, CKD, hemoptysis, alteration of taste, and immunosuppressive therapy were associated with higher odds in the vaccinated group.

As a result of our approach, which we believe is the first of its kind, at least one immunization dose reduces risk severity. In addition, various predictors, such as older age, presence of comorbidities, smoking, and hemoptysis, are associated with severity in vaccinated patients. However, the mechanism by which partial vaccination reduces the severity of the illness remains largely unknown. 

Our study has some limitations. It was a single-center, observational study with small sample size and short duration. There is also a scarcity of information about the type of vaccines, variations, and their impact on illness severity. Our study included fewer vaccinated patients, although it still showed valuable results.

## Conclusions

Most patients with COVID-19 admitted to the ICU in our study were non-vaccinated. Most partially vaccinated patients got infected before developing immunity, and a small percentage of completely vaccinated patients were likely non-responders. Vaccinated patients had a less severe illness, more comorbidities, fewer inflammatory markers, and less mortality. Age >60 years and HTN, DM, ILD, and smoking were associated with the worst outcome in vaccinated patients.
